# High Temperature Terahertz Detectors Realized by a GaN High Electron Mobility Transistor

**DOI:** 10.1038/srep46664

**Published:** 2017-04-21

**Authors:** H. W. Hou, Z. Liu, J. H. Teng, T. Palacios, S. J. Chua

**Affiliations:** 1 Low-energy electronic system IRG, Singapore-MIT Alliance for Research and Technology Center, 1 CREATE Way, 138602, Singapore; 2 Department of Electrical and Computer Engineering, National University of Singapore, Block E4, Engineering Drive 3, 117583, Singapore; 3 Institute of Materials Research and Engineering, Agency for Science, Technology, and Research (A*STAR), 2 Fusionopolis Way, Innovis, 138634, Singapore; 4 Department of Electrical Engineering and Computer Science, Massachusetts Institute of Technology, 77 Massachusetts Avenue, Cambridge, Massachusetts, 02139, United States

## Abstract

In this work, a high temperature THz detector based on a GaN high electron mobility transistor (HEMT) with nano antenna structures was fabricated and demonstrated to be able to work up to 200^ ^°C. The THz responsivity and noise equivalent power (NEP) of the device were characterized at 0.14 THz radiation over a wide temperature range from room temperature to 200 °C. A high responsivity *R*_v_ of 15.5 and 2.7 kV/W and a low NEP of 0.58 and 10 pW/Hz^0.5^ were obtained at room temperature and 200 °C, respectively. The advantages of the GaN HEMT over other types of field effect transistors for high temperature terahertz detection are discussed. The physical mechanisms responsible for the temperature dependence of the responsivity and NEP of the GaN HEMT are also analyzed thoroughly.

With the development of terahertz (THz) technologies, a compact, high speed, and highly sensitive detector working at sub-THz and THz ranges is highly desirable^1^,^2^,^3^,^4^,^5^,^6^. Field-effect transistors (FETs) have been used to fabricate THz detectors, benefiting from the nonlinear properties of the plasmonic excitations in the two-dimensional electron channels. Field-effect transistor THz detectors hold the advantages of high sensitivity, fast response, ability to operate at room temperature^7^. Different types of FETs, such as silicon metal-oxide-semiconductor FETs (MOSFETs), GaAs high electron mobility transistors (HEMTs), InP HEMTs, or GaN HEMTs have been reported with broadband responsivities for THz radiation^8^. For example, the responsivity of Si-FET integrated with a bow-tie antenna has reached 5 kV/W at 0.292 THz^9^. A very high responsivity of 22.7 kV/W at 0.2 THz has been demonstrated using an InP HEMT with an asymmetric dual-grating-gate^7^. GaN HEMTs integrated with a nano-antenna have shown a room temperature responsivity of 15 kV/W at 0.14 THz in our previous work^10^.

It is demanding to develop terahertz detectors with high sensitivity at elevated temperatures for applications in a harsh environment. The currently widely used terahertz detectors are the bolometer, the pyroelectric detector, the Schottky diode, and Golay cells. Bolometers only work well at low temperatures. The pyroelectric detector and Golay cells detect terahertz radiation up to room temperature^11^. This is due to their intrinsic limitation that detection is based on the induced temperature change by the absorption of terahertz photons and a higher temperature will increase the background noise and reduce sensitivity. Schottky diode or FET detectors made of Si, GaAs, or InP have different detection mechanisms and are able to work at room temperature. However, Si, GaAs, or InP has a relatively narrow band gap (1.12~1.42 eV), and thereby a high intrinsic carrier density that increases exponentially with temperature, which will enlarge the device leakage current and degrade the device performance. Therefore, it is difficult for Si, GaAs, or InP FET terahertz detectors to work at high temperatures.

As a wide-bandgap material, GaN has attracted much interest from both academia and industry for electronic^12^ and optoelectronic device applications^13^. GaN HEMTs have also shown excellent performance for sub-THz and THz detection. Panasonic Corp. has reported a detection responsivity of 1.1 kV/W at 1 THz using a GaN HEMT with 80 nm gate dipole antennas^14^. With a large bandgap of GaN at ~3.4 eV and thus lower intrinsic carrier density, GaN HEMTs are endowed with the potential to work well at high temperatures^15^.

In this paper, we report for the first time a THz detector based on GaN HEMT with the ability to work at high temperatures up to 200 °C. It is the first semiconductor device allowing terahertz detection that can operate at such a high temperature. The mechanisms inside the temperature dependence of the detection responsivity and the noise equivalent power (NEP) are also studied.

## Advantages of GaN HEMTs for High Temperature Terahertz Detection

A fundamental theoretical description of the non-resonant THz detection in FETs has been given in the framework of the Dyakonov–Shur theory^16^. The induced terahertz detection signal Δ*U* can be found from


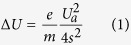


where *s* is the plasma wave velocity. The plasma wave velocity *s* is given by^17^


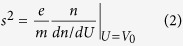


By adding intrinsic carrier density *n*_i_ to the unified charge control model^18^, the electron density *n* in the 2DEG channel of a HEMT can be described by:


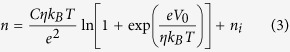


Here, *V*_0_ is the gate-to-channel voltage swing defined as *V*_0_ = *V*_g_ − *V*_th_, *C* = *en*_0_/, *V*_th_ is the gate capacitance, *η* is the ideality factor, *k*_*B*_ is the Boltzmann constant, *T* is the absolute temperature, and *n*_i_ is the intrinsic carrier density of the semiconductor.

The intrinsic carrier density *n*_i_ of semiconductors can be determined by^19^:


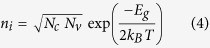


It can be seen that the intrinsic carrier density is exponentially dependent on the bandgap, which is a huge difference for semiconductors. *N*_*c*_ and *N*_*v*_ are the effective density of states in the conduction band and the valence band. In a bulk semiconductor, *N*_*c*_ and *N*_*v*_ are proportional to *T *^3/2^. In a 2DEG system, as the energy level s are quantized and most of the electrons (more than 80%) occupy the first level^20^, the influence of the temperature on *N*_*c*_ and *N*_*v*_ is very weak. Considering that the THz detection performance of a HEMT is much higher when it is operating in 2DEG depletion region than in accumulation region, we use 3D bulk temperature dependence for *N*_*c*_ which is approximately proportional to *T *^3/2^, instead of the 2D temperature dependence.

The intrinsic carrier density for silicon is about ~1 × 10^10^ cm^−3^. For GaAs, it is about ~2 × 10^6^ cm^−3^, while for InP, it is about 1 × 10^7^ cm^−3^. However, for GaN, the intrinsic carrier density is extremely low (~1.9 × 10^−10^ cm^−3^) due to its wide bandgap, which will provide GaN unique properties for high temperature terahertz detection.

The temperature dependence of the band gap is calculated by the Varshni equation. Using Eqs (2) and (3), we get


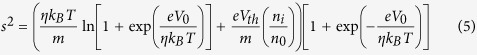


Combining Eqs (1) and (5), the terahertz response Δ*U* can be expressed as


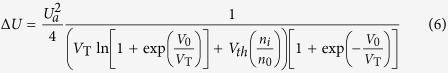


where *V*_T_ = *ηk*_*B*_*T*/*e* and is related to the sub-threshold slope; *n*_0_ is the electron density in the channel (which is 1 × 10^13^ cm^−2^ for GaN HEMTs, 7 × 10^11^ cm^−2^ for GaAs HEMTs^21^, 1 × 10^13^ cm^−2^ for InP HEMTs^22^, and 3.5 × 10^11^ cm^−2^ for silicon MOSFET^3^); and *U*_*a*_ is the coupling efficiency of the incoming radiation to the antenna.

**Figure 1:**
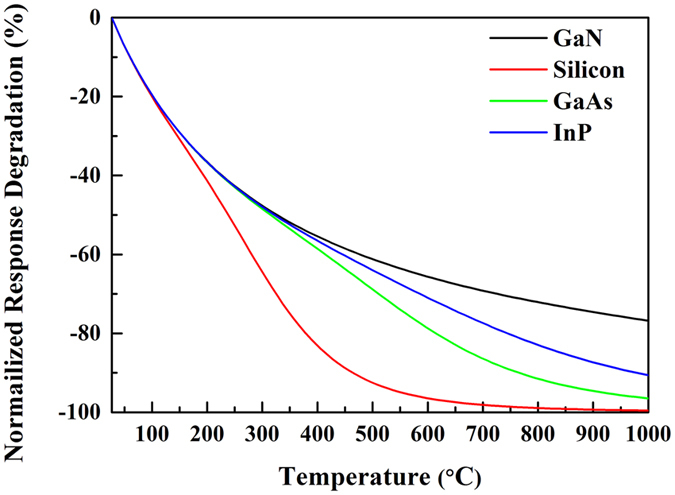
The normalized response degradation (Δ*U*(T) − Δ*U*(300 K))/Δ*U*(300 K) with temperature for GaN HEMTs, GaAs HEMTs, InP HEMTs and Silicon MOSFET.

The normalized response degradation (Δ*U*(T) − Δ*U*(300 K))/Δ*U*(300 K) with temperature for GaN HEMTs, GaAs HEMTs, InP HEMTs, and silicon MOSFETs is plotted in Fig. 1. At room temperature, the intrinsic carrier concentration is negligible compared to *n*_0_ and the ratio *n*_i_/*n*_0_ « 1 for all the transistors. Therefore, the term *V*_th_(*n*_i_/*n*_0_) is negligible and Eq. (6) is reduced to the well-established equation used at room temperature^17^. In a low temperature region, all types of transistors decrease with *T*^−1^, which is mainly a result of the *V*_T_ increment. When the temperature >100 °C, the intrinsic carrier concentration *n*_i_ in silicon MOSFETs starts to influence the device performance so that the response degradation for silicon MOSFET reduces faster than in other type of FETs. As the temperature increases, the degradation of GaAs HEMTs and InP HEMTs speed up as well. It can be seen GaN HEMTs perform the best over other transistors, even up to 1000 °C. At 1000 °C, the intrinsic carrier concentration in GaN is only 3.86 × 10^9^ cm^−2^. It is still much lower than the channel concentration of 1 × 10^13^ cm^−2^, which indicates that the intrinsic carrier concentration little influence on GaN HEMTs. Another important finding is that the ultra-low intrinsic carrier concentration of GaN due to its 3.4 eV wide bandgap and its high channel carrier concentration of 1 × 10^13^ cm^−2^ make GaN HEMTs suitable for high temperature terahertz detection.

Another advantage of GaN HEMTs is their low subthreshold leakage at high temperature^23^. The subthreshold leakage is able to induce shot noise (*N*_shot_ = (2e*I*_leakge_)^0.5^) for the terahertz detector. The wide band gap helps to suppress subthreshold leakage for the GaN terahertz detector.

## Demonstration of High Temperature GaN Terahertz Detector

**Figure 2:**
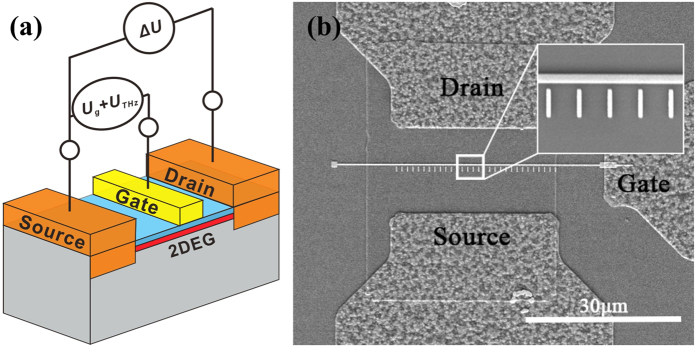
(**a**) The schematic diagram of GaN HEMT terahertz detector. (**b**) SEM image of the GaN HEMT terahertz detector from top view.

The detector schematic is as shown in Fig. 2(a). The device fabricated is based on an Al_0.25_Ga_0.75_N/GaN heterostructure. The epilayers include a 3 nm unintentionally-doped (UID) GaN cap, a 20 nm UID Al_0.25_Ga_0.75_N barrier, a 1 nm AlN spacer, a 1.5 μm GaN buffer layer, and transition/seeding layers. The epilayers were grown on a 6-inch high-resistivity Si(111) substrate. The typical 2-dimensional electron gas (2DEG) mobility and density are ~2100 cm^2^/Vs and ~1.0 × 10^13^ cm^−2^, respectively, as measured by the Hall measurement.

The scanning electron microscopy (SEM) image of the top view of the THz detector is shown in Fig. 2(b). An asymmetric nano antenna was placed near the gate of the HEMT. The gate length is 250 nm and the nano antenna has the dimension of 100 nm × 1 μm. The gap between the gate and the nano antenna is 200 nm. The source-drain distance is 14 μm. The nano-antenna was designed to improve the coupling between the incident THz radiation and the plasmons in the 2DEG channel^10^.

**Figure 3:**
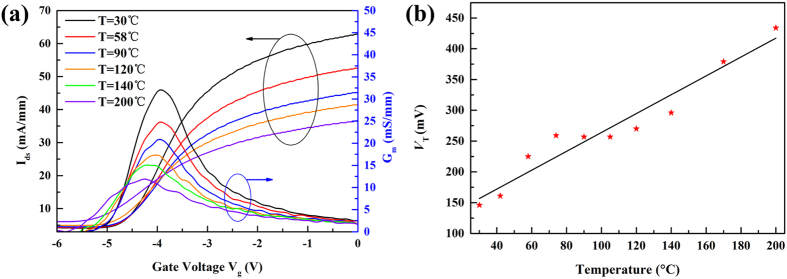
(**a**) The transfer characteristics of the GaN HEMT. (**b**) Temperature dependent modified thermal voltage *V*_T_, obtained from transfer characteristics measurements.

The transfer current-voltage characteristics were measured from room temperature to 200 °C, and they are shown in Fig. 3(a). The fabricated GaN devices exhibit good gate modulation and transfer properties. It can be seen that source-drain current decreases from 31 mA/mm at 30 °C to 17 mA/mm at 200 °C with *V*_g_ = 0 V and *V*_d_ = 1 V. This is mainly due to the mobility degradation at high temperature^20^. When the temperature is more than −173 °C, the 2DEG channel mobility is predominantly limited by the optical phonon scattering^24^. In addition, at higher temperature, the transconductance *G*_*m*_ is reduced, the curve of *G*_*m*_ versus *V*_g_ is broadened, and the *G*_*m*_ peak position is shifted. The broadening is due to the change of the thermal voltage *V*_*T*_, which has its relationship with temperature as shown in Fig. 3(b), and to the ideal factor*η*^18^. The threshold voltage *V*_*th*_ is −4.5 V at room temperature and it shifts to a more negative voltage of −5.5 V at 200 °C, which is a typical behavior for the GaN HEMT^25^.

**Figure 4:**
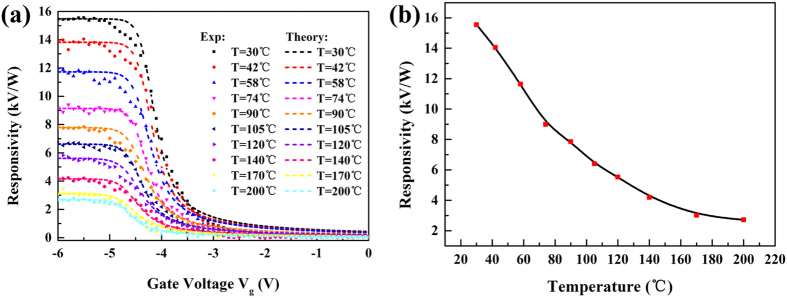
(**a**) Responsivity measured as a function of gate voltage at different temperatures for radiation at 0.14 THz. (**b**) Temperature dependent responsivity at *V*_g_ = −5.5 V.

The responsivity *R*_*v*_ as a function of the gate voltage (*V*_g_) at different temperatures from room temperature to 200 °C is plotted in Fig 4(a). The sigmoid shapes of the responsivity versus gate bias are similar at all measured temperatures up to 200 °C. *R*_v_ starts to decrease when *V*_g_ > *V*_th_, and it is close to zero when *V*_g_ > > *V*_th_. When *V*_g_ decreases and enters the depletion regime (*V*_g_ = −3 to −5 V, around *V*_th_), *R*_v_ starts to increase and saturates after the 2DEG channel is fully depleted (*V*_g_ < *V*_th_).

As seen from Fig. 4(a), the responsivity decreases with the increase of temperature at all gate biases. The measured maximum responsivity at 0.14 THz was around 15 kV/W at room temperature, and it decreased by five times to 2.7 kV/W at 200 °C, as shown in Fig. 4(b). This decrease shows the excellent performance of the GaN HEMT to work as a THz detector at high temperatures. In addition, the position of the maximum THz responsivity negatively shifted as the temperature increased. This shift is due to the threshold voltage shift of the GaN HEMT, which is a typical behavior of a GaN HEMT^25^.

Without the influence of an intrinsic carrier, the term determining the maximum THz response for GaN HEMTs mainly includes the modified thermal voltage *V*_T._ As for the term *V*_T_, due to the temperature dependence of *η*, the relationship between *V*_T_ and temperature *T* is not linear. The temperature dependence of *V*_T_ can be seen from the sub-threshold slope of the transfer characteristics of the GaN HEMT^26^. By analyzing the experimental transfer characteristics (Fig. 3(a)), the temperature dependence of *V*_T_ for our investigated transistor can be determined and plotted as shown in Fig. 3(b). It can be seen that the subthreshold slope decreases and the *V*_T_ increases with increasing temperature. According to Eq. (6), the detected voltage Δ*U* is inversely proportional to *V*_T_, so it will become smaller at a higher temperature.

**Figure 5:**
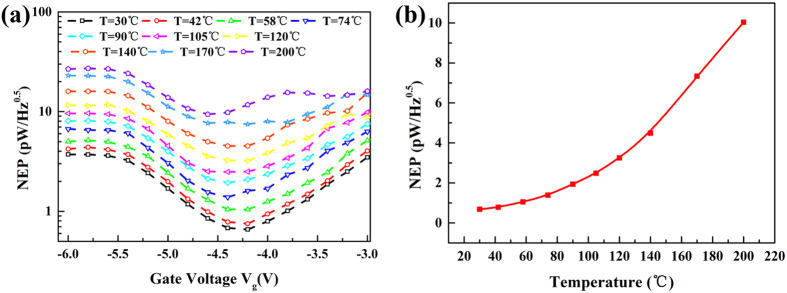
(**a**) NEP of the GaN HEMT detector as a function of gate voltage at different temperatures. (**b**) NEP of the GaN HEMT detector as a function of temperature at gate voltage *V*_g_ = −4.4 V.

Noise equivalent power is an important figure of merit for a THz detector. The dominant noise source in the GaN terahertz detector is the thermal noise from the GaN channel. Therefore, the NEP can be estimated by Eq. (7):


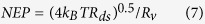


where *R*_*ds*_ is the channel resistance. *R*_*ds*_ can be determined from the static transfer characteristic shown in Fig. 3(a). Figure 5 shows the NEP value as a function of gate voltage from room temperature to 200 °C. Because of the additional factor *R*_ds_ in Eq. (7), the shape of the NEP versus the gate voltage does not follow that of *R*_v_. Due to the sharp rise of the channel resistivity, the NEP curve at *V*_g_ < *V*_th_ shows a valley as indicated in Fig. 5(a). The increase of temperature leads to the increase of the thermal noise and thus the NEP. A minimal NEP value of 0.58 pW/Hz^0.5^ is obtained at *V*_g_ = −4.2 V at room temperature, and 9.38 pW/Hz^0.5^ is obtained at *V*_g_ = −4.6 V at 200 °C. Fig. 5(b) shows the temperature dependence of the NEP at the fixed gate voltage *V*_g_ = −4.4 V. Compared with the degradation of the responsivity *R*_v_ at high temperatures, the physical mechanisms inside the degradation of the NEP include an extra factor of thermal noise, which is proportional to *T *^0.5^. To achieve higher operation temperature, surface states of GaN HEMTs can be passivated to enhance device thermal stability^27^,^28^.

## Conclusion

In conclusion, a THz detector working up to 200 °C was successfully demonstrated based on a nano-antenna GaN HEMT. A maximum responsivity 15 kV/W and 2.7 kV/W and a minimum NEP of 0.58 pW/Hz^0.5^ and 9.38 pW/Hz^0.5^ were achieved at 0.14 THz radiation at room temperature and 200 °C, respectively. The low intrinsic carrier concentration, high channel carrier concentration, and low subthreshold leakage were found to be advantages of the GaN terahertz detector at high temperatures. The excellent performance of the GaN THz detector manifests itself as a promising candidate for high temperature THz applications.

## Methods

### Device Fabrication

The fabrication process started from mesa patterning and isolation by Cl_2_ based plasma etching. Source and drain ohmic contacts were formed with Ti/Al/Ni/Au (20/120/40/50 nm) by e-beam evaporation followed by 850 °C annealing in an N_2_ atmosphere. The gate was realized by Ni/Au (20/80 nm) metallization. The surface was not passivated.

### Experimental Setup

A 0.14 THz (full name) IMPATT diode was used as a continuous-wave (CW) sub-THz source with the output power of 0.1 mW, which was calibrated by a standard pyroelectric power meter. The THz radiation was focused onto the surface of the device with a beam spot size of around 1.5 mm in diameter by two off-axis parabolic mirrors. The drain terminal of the HEMT was not biased, and the gate terminal was biased through a DC power source (GWInstek GPD-3303S). The photovoltages Δ*U* generated at the drain-source terminal were read out by a SR830 lock-in amplifier. A 10 mm × 10 mm metal ceramic heater was attached on the backside of the device and a thermocouple was placed on the front side of the device to measure the device temperature. The responsivity *R*_*v*_ is defined as Δ*U*/*P*_*d*_, where Δ*U* is the measured photovoltage and *P*_*d*_ is the radiation power on the detector.

## Additional Information

**How to cite this article:** Hou, H. W. *et al*. High Temperature Terahertz Detectors Realized by a GaN High Electron Mobility Transistor. *Sci. Rep.* **7**, 46664; doi: 10.1038/srep46664 (2017).

**Publisher's note:** Springer Nature remains neutral with regard to jurisdictional claims in published maps and institutional affiliations.
